# Association between thyroid hormone sensitivity indices and surrogate clinicopathologic subtypes of breast cancer: A retrospective single-center study

**DOI:** 10.1097/MD.0000000000051340

**Published:** 2026-10-30

**Authors:** Mehmet Mert Hidiroglu, Sevket Baris Morkavuk, Safak Akin, Ibrahim Burak Bahcecioglu, Nese Ersoz Gulcelik, Mehmet Ali Gulcelik

**Affiliations:** a Department of General Surgery, Health Sciences University, Faculty of Medicine, Gülhane Research and Training Hospital, Ankara, Turkey; b Department of Surgical Oncology, Health Sciences University, Faculty of Medicine, Gülhane Research and Training Hospital, Ankara, Turkey; c Department of Endocrinology and Metabolism, Health Sciences University, Faculty of Medicine, Gülhane Research and Training Hospital, Ankara, Turkey.

**Keywords:** breast cancer, heterogeneous disease, surrogate clinicopathologic subtype, thyroid hormone sensitivity index

## Abstract

Despite advances in screening and treatment for breast cancer and a substantial decline in the disease’s mortality, breast cancer continues to show increased incidence and remains a major global health burden. A major factor in these trends is breast cancer’s heterogeneity, as reflected by distinct subtypes. Central thyroid hormone sensitivity can be assessed using the thyroid feedback quantile-based index (TFQI), thyroid-stimulating hormone (TSH) index (TSHI), TSH T4 resistance index (TT4RI), and the free triiodothyronine/free thyroxine (fT3/fT4) ratio, an indirect proxy of peripheral thyroid hormone conversion. We investigated associations between surrogate clinicopathologic subtypes of breast cancer and central thyroid hormone sensitivity indices and the fT3/fT4 ratio. We retrospectively analyzed 109 patients with preoperative thyroid function tests who underwent surgery for breast cancer between 2017 and 2022 at Gülhane Training and Research Hospital, Department of Surgical Oncology. We investigated associations of surrogate clinicopathologic subtypes with TFQI, TT4RI, TSHI, and the fT3/fT4 ratio. Associations with receptor and Ki-67 status were additionally evaluated using separate age- and BMI-adjusted Firth’s penalized logistic regression models, each including 1 thyroid index. No statistically significant differences emerged in the distribution of central thyroid hormone sensitivity indices across surrogate clinicopathologic subtypes (*P* = .349 for TFQI, *P* = .867 for TT4RI, *P* = .645 for TSHI). The fT3/fT4 ratio differed significantly across subtypes (*P* < .001). In separate age- and BMI-adjusted Firth’s models, a higher fT3/fT4 ratio was associated with lower odds of HER2 positivity (adjusted OR = 0.226, 95% CI: 0.088–0.579; *P* = .002) but higher odds of being in the high Ki-67 group (adjusted OR = 2.096, 95% CI: 1.219–3.603; *P* = .007). No statistically significant associations were observed between these outcomes and central thyroid hormone sensitivity indices. The fT3/fT4 ratio differed significantly across breast cancer subtypes, with higher values in the triple-negative breast cancer group than in several other subtypes, whereas central thyroid hormone sensitivity indices showed no significant differences. These findings suggest a possible association between peripheral thyroid hormone conversion and clinicopathologic tumor phenotype.

## 1. Introduction

Breast cancer accounts for approximately 32% of all newly diagnosed cases of cancer in the female population.^[1]^ While a woman’s lifetime risk of developing breast cancer is 1 in 8, a woman’s likelihood of dying from it is 1 in 39.^[2]^ With advances in medicine, increased knowledge about and experience with breast cancer’s etiology, and the development of new screening and treatment methods, guidelines on the mechanisms of the disease’s formation and the algorithm for its treatment are changing by the day. However, despite advances in the screening and treatment of the disease and a substantial decline in its mortality, breast cancer has continued to show increased incidence and remains a major global health burden. Along those lines, a major challenge in combating breast cancer is its biological heterogeneity, which contributes to variability in tumor behavior, prognosis, and responses to treatment.^[3]^

Found in Egypt, the first records of breast cancer date to roughly 3500 BC.^[4]^ In the 18th century, Valsalva first drew attention to the axillary lymph nodes, and in the late 19th century, surgery for breast cancer gained momentum when Halsted described radical mastectomy.^[5]^ That development was quickly followed by modified radical mastectomy, breast-conserving surgery, sentinel lymph node sampling, and oncoplastic breast surgery (OBS). In such processes, the most important factor in protecting the breast is determining the disease’s risk factors and the factors that largely affect the prognosis. However, increased rates of breast cancer recurrence in recent years, especially in patients without axillary lymph node metastases or different long-term results of patients with equivalent tumor stages and in the same histopathological group, now compel researchers to investigate other prognostic factors for the disease. In that context, an important milestone toward understanding breast cancer’s heterogeneity has been its gene-expression-based molecular classification introduced by Perou et al, who in 2000 identified distinct intrinsic molecular subtypes of breast cancer using complementary DNA microarrays.^[6]^ In routine clinical practice, those intrinsic subtypes are commonly approximated using surrogate clinicopathologic classifications based on estrogen receptor (ER), progesterone receptor (PR), HER2, and Ki-67 status. That classification has contributed substantially to the understanding of breast cancer’s biological heterogeneity.

Standard thyroid function tests evaluate free triiodothyronine (fT3), free thyroxine (fT4), and thyroid-stimulating hormone (TSH) levels. However, beyond those standard measurements, thyroid hormone sensitivity can be evaluated using central indices, including the thyroid feedback quantile-based index (TFQI), TSH index (TSHI), and thyrotrophT4 resistance index (TT4RI), while the fT3/fT4 ratio is used as an indirect proxy of peripheral thyroid hormone conversion.^[7,8]^ The principal logic of those indices is based on the relationship of thyroid hormone with feedback regulation in the central system and resistance in the peripheral target organ. Recent studies have shown that any disruption in the individual or coordination of those 2 regulatory systems is also associated with the presence of certain endocrine diseases, including diabetes mellitus, obesity, metabolic disease, and renal failure.^[9]^

To date, studies have suggested an association between thyroid hormone status and breast cancer, with higher circulating levels of thyroid hormone being associated with the risk of breast cancer even within the euthyroid range.^[10]^ Several biological mechanisms may underlie that relationship because thyroid hormone can influence the proliferation and survival of breast cancer cells and may interact with ER signaling.^[11]^ However, the relationship between thyroid hormone sensitivity indices and surrogate clinicopathologic subtypes of breast cancer remains poorly understood. Therefore, evaluating those indices may provide additional insight into the relationship between thyroid hormone signaling and the clinicopathologic characteristics of breast cancer.

In our study, we investigated the associations of central thyroid hormone sensitivity indices and the fT3/fT4 ratio with surrogate clinicopathologic subtypes of breast cancer. We restricted the study to euthyroid patients to investigate these associations within the normal range of thyroid function. We also evaluated their associations with ER, PR, HER2, and Ki-67 status, which were used to define the surrogate clinicopathologic subtypes.

## 2. Materials and methods

### 2.1. Study population

The medical records of patients who underwent surgery for breast cancer between 2017 and 2022 in the Department of Surgical Oncology, Faculty of Medicine, Health Sciences University, Gülhane Training and Research Hospital, were retrospectively reviewed. A total of 145 patients with complete preoperative TSH, fT3, and fT4 measurements were identified. Exclusion criteria were applied sequentially. First, 8 patients with a history of thyroidectomy were excluded, leaving 137 patients. Subsequently, 28 patients with non-euthyroid thyroid function and/or thyroid-related medication use were excluded, leaving 109 euthyroid patients in the final study cohort. Euthyroid status was defined as serum TSH, fT3, and fT4 levels within the institutional laboratory reference ranges (TSH: 0.35–5.33 mIU/L; fT3: 2.1–4.6 pg/mL; fT4: 0.58–1.44 ng/dL). Because all 145 patients had complete thyroid measurements, no patients within this group were excluded for missing TSH, fT3, or fT4 values. Each excluded patient was counted only once in the sequential selection process (Fig. 1).

**Figure 1.**
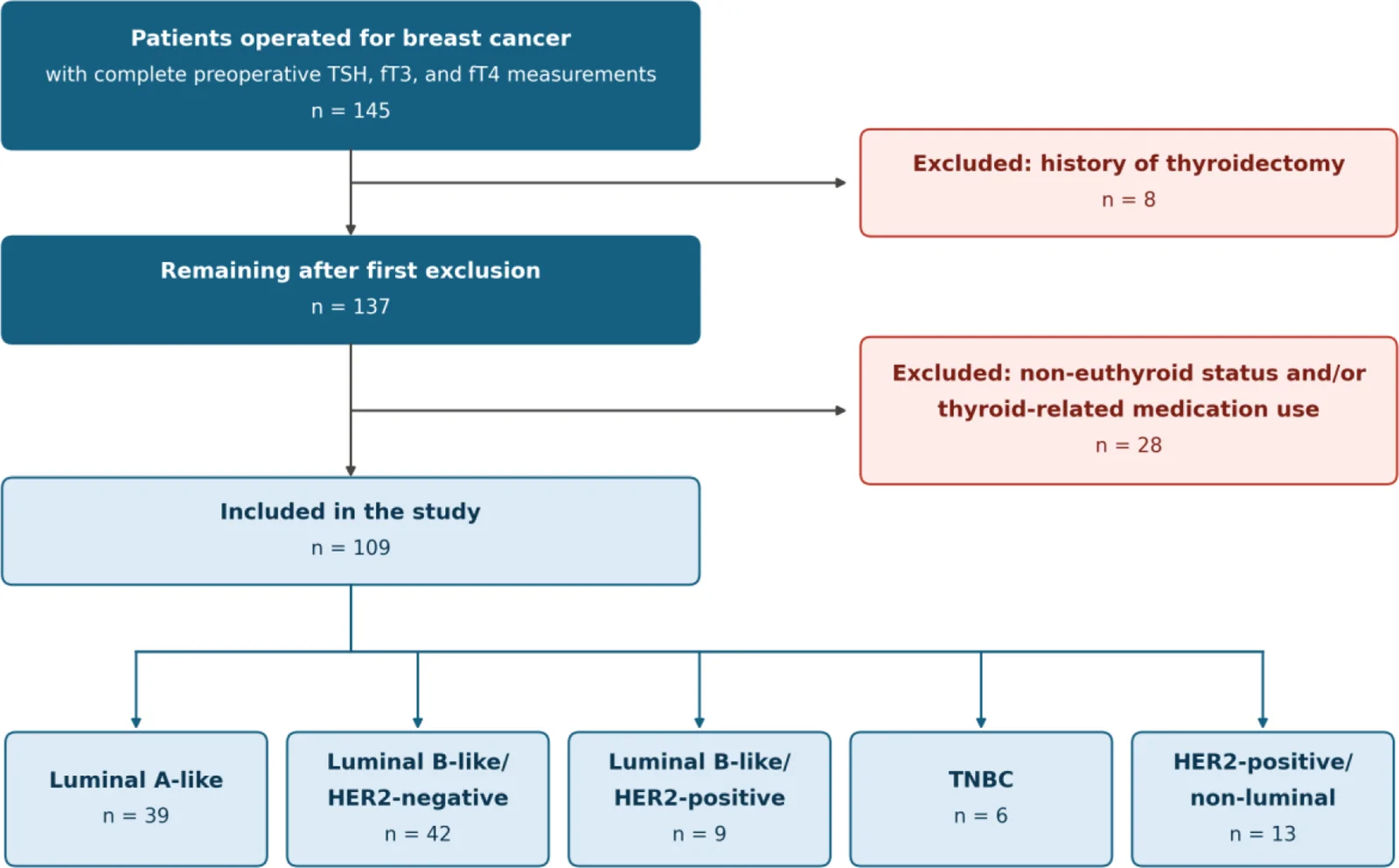
Study flow diagram. fT3 = free triiodothyronine, fT4 = free thyroxine, TNBC = triple-negative breast cancer, TSH = thyroid-stimulating hormone.

Our study was performed in accordance with the Declaration of Helsinki and reviewed and approved by the Ethics Committee of the Ankara Gülhane Research and Training Hospital (Date: 12.09.2022, No. 2022-277).

### 2.2. Calculation of thyroid hormone sensitivity indices and definition of surrogate clinicopathologic subtypes

Central thyroid hormone sensitivity indices included the TFQI, TSHI, and TT4RI, whereas the fT3/fT4 ratio was evaluated as an indirect proxy of peripheral thyroid hormone conversion. Serum fT4 values, originally reported by our institutional laboratory in ng/dL, were converted to pmol/L using the conversion factor 12.87. TSHI was calculated as ln[TSH (mIU/L)] + 0.1345 × fT4 (pmol/L), in which ln denotes the natural logarithm. TT4RI was calculated as fT4 (pmol/L) × TSH (mIU/L), and TFQI was calculated as cdf(fT4) − [1 − cdf(TSH)], in which cdf denotes the cumulative distribution function of the corresponding variable. Lastly, the fT3/fT4 ratio was calculated by directly dividing the laboratory-reported fT3 value in pg/mL by the fT4 value in ng/dL.^[7,8,12]^ As an example, for a participant with a TSH level of 3.770 mIU/L and an fT4 level of 1.12 ng/dL, fT4 was first converted to 14.41 pmol/L, after which TSHI was calculated as ln(3.770) + 0.1345 × 14.41 = 3.27 and TT4RI as 14.41 × 3.770 = 54.34.

ER, PR, HER2, and Ki-67 results were obtained from the final pathology reports. ER and PR were assessed using immunohistochemistry and were considered positive when nuclear staining was present in ≥1% of invasive tumor cells. HER2 immunohistochemical staining was scored as 0, 1+, 2+, or 3+ in accordance with the applicable criteria of the American Society of Clinical Oncology and the College of American Pathologists. Scores of 0 and 1+ were considered negative, whereas a score of 3+, defined by complete and intense circumferential membrane staining in >10% of invasive tumor cells, was considered positive. Cases with an equivocal HER2 score of 2+ underwent confirmatory in situ hybridization, and final HER2 status was determined according to the presence or absence of HER2 gene amplification. Lastly, the Ki-67 proliferation index was recorded as the percentage of positively stained invasive tumor cell nuclei reported in the final pathological assessment. A prespecified cutoff of 14% was used to categorize Ki-67 as low (<14%) or high (≥14%) for the classification of surrogate clinicopathologic subtype. The cutoff was selected according to the St. Gallen 2013 surrogate classification for breast cancer and not derived from the outcomes in our study’s cohort.

Meanwhile, surrogate clinicopathologic subtype was classified according to the St. Gallen 2013 Consensus criteria using ER, PR, HER2, and Ki-67 status. Patients were classified into 5 groups: luminal A-like (ER-positive, PR ≥ 20%, HER2-negative, and Ki-67 < 14%); luminal B-like/HER2-negative (ER-positive, HER2-negative, and Ki-67 ≥ 14% and/or PR < 20%); luminal B-like/HER2-positive (ER- and/or PR-positive and HER2-positive, irrespective of Ki-67); HER2-positive/non-luminal (ER-negative, PR-negative, and HER2-positive); and triple-negative breast cancer (TNBC; ER-negative, PR-negative, and HER2-negative).^[13]^

### 2.3. Study design

Clinical and demographic data were obtained by retrospectively examining patients’ medical records. Preoperative thyroid function tests obtained during routine preoperative evaluation were used to calculate the central thyroid hormone sensitivity indices and the fT3/fT4 ratio as an indirect proxy of peripheral thyroid hormone conversion. First, we examined the associations between thyroid hormone sensitivity indices and the surrogate clinicopathologic subtypes of breast cancer. Afterward, we compared the thyroid hormone sensitivity indices across those subtypes and subsequently evaluated the associations of the relevant indices with ER, PR, HER2, and Ki-67 status. Last, exploratory receiver operating characteristic (ROC) analyses were used to evaluate cohort-specific fT3/fT4 ratio thresholds for HER2 and Ki-67 status.

### 2.4. Statistical analyses

Statistical analyses were performed using IBM SPSS Statistics version 22.0 (IBM Corp, Armonk) and Python version 3.12.14 (Python Software Foundation, Wilmington). Firth’s penalized logistic regression models and Dunn’s post hoc pairwise comparisons were performed in Python. All variables included in the analyses were complete for the study’s final cohort, and no missing data were imputed. The normality of thyroid hormone sensitivity indices was assessed using the Shapiro–Wilk test, while categorical variables were compared using Pearson’s chi-square test or Fisher’s exact test, as appropriate. For comparisons of continuous variables between 2 groups, Student *t* test was used for normally distributed data and the Mann–Whitney *U* test for non-normally distributed data. For comparisons involving more than 2 groups, a 1-way analysis of variance (ANOVA) or the Kruskal–Wallis test was used, as appropriate. Following the significant Kruskal–Wallis test for the fT3/fT4 ratio, pairwise comparisons were performed using Dunn’s test with correction for tied ranks and Bonferroni adjustment across all 10 comparisons among the 5 subtypes; standardized z statistics and 2-sided adjusted *P* values are reported. TFQI, TT4RI, and TSHI were analyzed as continuous variables, and no predefined cutoff values were applied to those indices. Univariable binary logistic regression analyses were performed separately for each candidate factor and each outcome (i.e., HER2, ER, PR, and Ki-67 status). Candidate factors included age, body mass index (BMI), comorbidity, smoking status, nodal metastasis, TSH, TSHI, TT4RI, TFQI, and fT3/fT4 ratio. For adjusted analyses, 4 separate multivariable Firth’s penalized logistic regression models were fitted for each outcome, each including 1 thyroid index together with age and BMI. Age and BMI were retained regardless of their univariable *P* values because age is associated with the physiology of the hypothalamic–pituitary–thyroid axis and menopausal status, whereas BMI may influence peripheral thyroid hormone metabolism and the adiposity-related endocrine milieu relevant to breast cancer. The thyroid indices were not entered simultaneously into the same model, in a parsimonious penalized approach used to limit model complexity and reduce small-sample estimation bias, particularly given the 18 HER2-positive cases. Multicollinearity was assessed using variance inflation factors, and odds ratios (ORs) for continuous variables were expressed per 1-unit increase. For Firth’s models, 95% confidence intervals (CIs) and 2-sided *P* values were calculated using Wald approximations. Exploratory ROC curve analyses were performed to evaluate the discrimination of HER2-negative status and high Ki-67 status by the fT3/fT4 ratio. Cohort-specific thresholds were selected by maximizing Youden’s index (sensitivity + specificity − 1) across candidate cutoffs defined as the midpoints between consecutive distinct observed values. Higher fT3/fT4 ratios indicated HER2-negative status or high Ki-67 status. These internally derived thresholds were not internally or externally validated and were not intended for clinical decision-making. The results of logistic regression analyses were expressed as ORs and 95% CIs. All analyses were considered exploratory. Except for the Bonferroni-adjusted Dunn post hoc comparisons, *P* values were not adjusted for multiple testing; *P* < .05 was used as the nominal significance threshold.

## 3. Results

### 3.1. Patients’ characteristics

In our study, 109 patients – all females – were evaluated retrospectively. The average age was 51.71 years (range: 27–85), average height 161.53 cm (range: 151–180), average weight 72.4 kg (range: 54–104), and average BMI 27.79 (range: 19.36–40.12). OBS plus sentinel lymph node biopsy (SLNB) was performed in 75 patients, axillary lymph node dissection after OBS+SLNB in 17 patients, and modified radical mastectomy in 17 patients. Among patients undergoing OBS + SLNB, 19 patients with 1 or 2 positive sentinel lymph nodes met the criteria for the omission of axillary lymph node dissection, consistent with the American College of Surgeons Oncology Group’s Z0011 approach and the American Society of Clinical Oncology’s current recommendations.^[14]^ As shown in Table 1, axillary lymph node metastasis was present in 53 patients (48.6%). Among the surrogate clinicopathologic subtypes, the luminal B-like/HER2-negative group, comprising 42 patients, was the most frequent. The luminal A-like, HER2-positive/non-luminal, luminal B-like/HER2-positive, and TNBC groups included 39, 13, 9, and 6 patients, respectively. ER was positive in 81.7% of patients, while PR was positive in 70.6%. The demographic and histopathological distribution of all patients appears in Table 1.

**Table 1:** Demographic and clinicopathological distribution of the patients.

Age, yr, mean ± SD (range)	51.71 ± 12.52 (27–85)
Height, cm, mean ± SD (range)	161.53 ± 5.58 (151–180)
Weight, kg, mean ± SD (range)	72.40 ± 10.32 (54–104)
BMI, kg/m^2^, mean ± SD (range)	27.79 ± 4.03 (19.36–40.12)
Type of surgical operation, n(%)	
OBS + SLNB	75 (68.8%)
OBS + ALND	17 (15.6%)
MRM	17 (15.6%)
Axillary lymp node metastasis	
Absent	56 (51.4%)
Present	53 (48.6%)
Estrogen receptor, n (%)	
Absent	20 (18.3%)
Present	89 (81.7%)
Progesterone receptor, n(%)	
Absent	32 (29.4%)
Present	77 (70.6%)
HER2 status, n(%)	
Negative	91 (83.5%)
Positive	18 (16.5%)
Ki-67 status, n (%)	
Low (<14%)	43 (39.4%)
High (≥14%)	66 (60.6%)
Surrogate clinicopathologic subtypes, n (%)	
Luminal A-like	39 (35.8%)
Luminal B-like/HER2-negative	42 (38.5%)
Luminal B-like/HER2-positive	9 (8.3%)
HER2-positive/non-luminal	13 (11.9%)
TNBC	6 (5.5%)
TSH, mIU/L, mean ± SD (range)	2.22 ± 1.08 (0.351–5.218)
fT3, pg/mL, mean ± SD (range)	3.11 ± 0.50 (2.11–4.57)
fT4, ng/dL, mean ± SD (range)	1 ± 0.20 (0.58–1.41)

ALND = axillary lymph node dissection, BMI = body mass index, MRM = modified radical mastectomy, OBS = oncoplastic breast surgery, SD = standard deviation, SLNB = sentinel lymph node biopsy, TNBC = triple-negative breast cancer, TSH = thyroid stimulating hormone.

### 3.2. Evaluation of thyroid hormone sensitivity indices

The mean TFQI was −0.20 ± 0.26 (range: −0.79 to 0.39), the mean TSHI was 2.38 ± 0.62 (range: 0.33–3.66), and the mean TT4RI was 27.72 ± 13.53 (range: 3.62–77.90). The median fT3/fT4 ratio was 3.07 (range: 1.73–5.40), as shown in Table 2.

**Table 2:** Central thyroid hormone sensitivity indices and the fT3/fT4 ratio in the overall study population.

TFQI, mean ± SD (range)	−0.20 ± 0.26 (−0.79 to 0.39)
TSHI, mean ± SD (range)	2.38 ± 0.62 (0.33–3.66)
TT4RI, mean ± SD (range)	27.72 ± 13.53 (3.62–77.90)
fT3/fT4 ratio, median (range)	3.07 (1.73–5.40)

Values are presented as mean ± SD (range), except for the fT3/fT4 ratio, which is presented as median (range) because of its non-normal distribution. Range denotes minimum–maximum.

fT3 = free triiodothyronine, fT4 = free thyroxine, SD = standard deviation, TFQI = thyroid feedback quantile-based index, TSHI = TSH index, TT4RI = thyrotroph T4 resistance index.

### 3.3. Distribution of thyroid hormone sensitivity indices across surrogate clinicopathologic subtypes

No statistically significant differences in TFQI, TT4RI, or TSHI emerged across the surrogate clinicopathologic subtypes (*P = *.349, *P = *.867, and *P = *.645, respectively). The fT3/fT4 ratio, however, did differ significantly across the surrogate clinicopathologic subtypes (*P < *.001). Dunn’s post hoc comparisons with Bonferroni adjustment showed significant differences between TNBC and the luminal A-like (adjusted *P *< .001), luminal B-like/HER2-positive (adjusted *P* = .008), and HER2-positive/non-luminal groups (adjusted *P *= .018). Significant differences were also observed between the luminal B-like/HER2-negative group and the luminal A-like (adjusted *P *< .001), luminal B-like/HER2-positive (adjusted *P* = .010), and HER2-positive/non-luminal groups (adjusted *P *= .031), as detailed in Table 3.

**Table 3:** Distribution of thyroid hormone sensitivity indices across surrogate clinicopathologic subtypes.

Variables	Surrogate clinicopathologic subtypes							*P* value
	Luminal A-like	Luminal B-like/HER2-negative		Luminal B-like/HER2-positive	Triple-negative breast cancer (TNBC)		HER2-positive/non-luminal	
TFQI, median (range)	−0.11 (−0.59 to 0.27)	−0.26 (−0.79 to 0.37)		−0.17 (−0.54 to 0.24)	−0.39 (−0.61 to 0.31)		−0.14 (−0.47 to 0.39)	.349*
TT4RI, median (range)	29.68 (3.62–55.40)	25.90 (6.87–77.90)		25.42 (13.78–57.45)	20.78 (9.92–49.38)		23.65 (12.35–47.44)	.867*
TSHI, median (range)	2.63 (0.33–3.54)	2.37 (0.93–3.66)		2.54 (1.80–3.23)	2.07 (1.31–3.30)		2.23 (1.66–3.30)	.645*
fT3/fT4, median (range)	2.58 (1.90–4.58)	3.58 (2.28–5.39)		2.75 (1.73–3.17)	4.09 (1.85–5.40)		2.62 (2.33–2.88)	**<.001** *

Post hoc pairwise comparisons of the fT3/fT4 ratio using Dunn’s test with Bonferroni adjustment								
Pairwise comparison			*Z*			Adjusted *P*		
TNBC vs Luminal A-like			4.240			**<.001**		
TNBC vs Luminal B-like/HER2-positive			3.353			**.008**		
TNBC vs HER2-positive/non-luminal			3.120			**.018**		
Luminal B-like/HER2-negative vs Luminal A-like			4.994			**<.001**		
Luminal B-like/HER2-negative vs Luminal B-like/HER2-positive			3.284			**.010**		
Luminal B-like/HER2-negative vs HER2-positive/non-luminal			2.962			**.031**		

Bold values indicate statistically significant *P* values (*P* < .05).

Post hoc comparisons used Dunn’s rank-based test with correction for tied ranks. *z* denotes the standardized test statistic for the first minus the second group. *P* values are 2-sided and Bonferroni-adjusted across all 10 pairwise comparisons. Only significant comparisons are shown.

fT3 = free triiodothyronine, fT4 = free thyroxine, TFQI = thyroid feedback quantile-based index, TNBC = triple-negative breast cancer, TSHI = TSH index, TT4RI = TSH T4 resistance index.

* Kruskal–Wallis test.

### 3.4. Association of the fT3/fT4 ratio with ER, PR, HER2, and Ki-67 status

Because the fT3/fT4 ratio differed significantly across the surrogate clinicopathologic subtypes, we further evaluated its associations with ER, PR, HER2, and Ki-67 status. In that process, because the fT3/fT4 ratio was not normally distributed, we used nonparametric tests. In the Mann–Whitney *U* test, no statistically significant difference in the fT3/fT4 ratio was observed according to ER or PR status (*P = *.15 and *P = *.252, respectively). However, we did find a statistically significant difference in the distribution of the fT3/fT4 ratio according to the Ki-67 index and HER2 receptor level groups (*P = *.001 and *P < *.001, respectively). The fT3/fT4 ratio was lower in HER2-positive patients than in HER2-negative patients (2.69 vs 3.36) and higher in patients with a high Ki-67 index than in patients with a low Ki-67 index (3.39 vs 2.87), as shown in Table 4. In univariable binary logistic regression analyses, each 1-unit increase in the fT3/fT4 ratio was associated with lower odds of HER2 positivity (OR = 0.203, 95% CI: 0.077–0.537; *P = *.001) and higher odds of high Ki-67 (OR = 2.206, 95% CI: 1.286–3.784; *P = *.004). No statistically significant associations were observed between the fT3/fT4 ratio and ER or PR status. Age, BMI, comorbidity, smoking status, nodal metastasis, TSH, TSHI, TT4RI, and TFQI were not significantly associated with any of the 4 outcomes in the univariable analyses (Table 5). In separate Firth’s penalized logistic regression models adjusted for age and BMI, each 1-unit increase in the fT3/fT4 ratio remained associated with lower odds of HER2 positivity (adjusted OR = 0.226, 95% CI: 0.088–0.579; *P = *.002) and higher odds of high Ki-67 (adjusted OR = 2.096, 95% CI: 1.219–3.603; *P = *.007). The corresponding associations with ER and PR status were not statistically significant (*P = *.284 and *P = *.430, respectively). None of the central thyroid hormone sensitivity indices showed a statistically significant association with any outcome in the adjusted models, and no evidence of problematic multicollinearity was observed (maximum variance inflation factor = 1.11), as shown in Table 6. In exploratory ROC analyses, the fT3/fT4 ratio yielded an AUC of 0.772 (95% CI: 0.682–0.861) for distinguishing HER2-negative from HER2-positive status. The threshold selected by maximizing Youden’s index was >3.04650, yielding 59.3% sensitivity and 94.4% specificity. For distinguishing high from low Ki-67 status, the AUC was 0.691 (95% CI: 0.589–0.794), and the corresponding threshold was >3.53253, yielding 47.0% sensitivity and 86.0% specificity (Fig. 2). These cohort-derived thresholds were not internally or externally validated and should not be interpreted as predictive or clinical decision thresholds.

**Table 4:** Association of the fT3/fT4 ratio with ER, PR, HER2, and Ki-67 status.

Variables	fT3/fT4 ratio, median (range)	*P* value
ER status		
Negative	3.55 (1.85–5.40)	.150*
Positive	3.00 (1.72–5.39)	
PR status		
Negative	3.24 (1.85–5.40)	.252*
Positive	3.02 (1.72–5.39)	
Ki-67 status		
Low	2.87 (1.90–5.39)	**.001** *
High	3.39 (1.72–5.40)	
HER2 status		
Negative	3.36 (1.85–5.40)	**<.001** *
Positive	2.69 (1.72–3.17)	

Bold values indicate statistically significant *P* values (*P* < .05).

Values are presented as median (minimum–maximum).

ER = estrogen receptor, fT3 = free triiodothyronine, fT4 = free thyroxine, HER2 = human epidermal growth factor receptor 2, PR = progesterone receptor.

* Mann–Whitney *U* test.

**Table 5:** Univariable binary logistic regression analyses of factors associated with hormone receptor status, HER2 status, and Ki-67 status.

Variables	Univariable analysis		
	*B*	OR (95% CI)	*P* value
Panel A. HER2 status			
Age (yr)	0.014	1.014 (0.975–1.055)	.479
BMI (kg/m^2^)	0.017	1.017 (0.898–1.152)	.792
Comorbidity (present)	0.113	1.120 (0.405–3.095)	.827
Smoking (present)	−0.535	0.586 (0.176–1.948)	.383
Nodal metastasis (present)	−0.474	0.623 (0.222–1.749)	.368
TSH (mIU/L)	−0.052	0.949 (0.590–1.527)	.829
TSHI	0.078	1.082 (0.474–2.466)	.852
TT4RI	−0.005	0.995 (0.958–1.034)	.807
TFQI	1.559	4.755 (0.681–33.218)	.116
fT3/fT4 ratio	−1.595	0.203 (0.077–0.537)	**.001**
Panel B. Ki-67 status			
Age (yr)	−0.015	0.985 (0.955–1.016)	.343
BMI (kg/m^2^)	−0.033	0.968 (0.879–1.065)	.501
Comorbidity (present)	0.445	1.561 (0.721–3.381)	.259
Smoking (present)	0.190	1.209 (0.527–2.773)	.654
Nodal metastasis (present)	−0.014	0.986 (0.457–2.126)	.971
TSH (mIU/L)	0.165	1.180 (0.819–1.700)	.375
TSHI	0.019	1.020 (0.549–1.895)	.951
TT4RI	0.002	1.002 (0.974–1.031)	.906
TFQI	−0.552	0.576 (0.130–2.539)	.466
fT3/fT4 ratio	0.791	2.206 (1.286–3.784)	**.004**
Panel C. Estrogen receptor status			
Age (yr)	0.039	1.040 (0.995–1.087)	.085
BMI (kg/m^2^)	0.022	1.023 (0.905–1.156)	.720
Comorbidity (present)	0.404	1.497 (0.565–3.969)	.417
Smoking (present)	0.063	1.065 (0.367–3.088)	.908
Nodal metastasis (present)	−0.313	0.731 (0.276–1.937)	.529
TSH (mIU/L)	0.084	1.088 (0.687–1.723)	.719
TSHI	0.312	1.366 (0.636–2.936)	.424
TT4RI	0.018	1.018 (0.980–1.058)	.364
TFQI	0.394	1.483 (0.222–9.899)	.684
fT3/fT4 ratio	−0.389	0.677 (0.385–1.191)	.176
Panel D. Progesterone receptor status			
Age (yr)	0.021	1.021 (0.987–1.058)	.230
BMI (kg/m^2^)	−0.021	0.979 (0.884–1.085)	.690
Comorbidity (present)	0.360	1.433 (0.627–3.278)	.394
Smoking (present)	0.460	1.584 (0.624–4.022)	.333
Nodal metastasis (present)	−0.255	0.775 (0.339–1.770)	.545
TSH (mIU/L)	−0.102	0.903 (0.619–1.318)	.598
TSHI	−0.010	0.990 (0.508–1.927)	.976
TT4RI	0.000	1.000 (0.970–1.031)	.997
TFQI	0.719	2.053 (0.403–10.460)	.387
fT3/fT4 ratio	−0.245	0.783 (0.481–1.275)	.326

Univariable binary logistic regression analyses were performed separately for each candidate factor and outcome. Odds ratios for continuous variables represent a one-unit increase. TSHI and TT4RI were calculated using fT4 in pmol/L, as described in the Methods. Bold values indicate *P* < .05.

*B* = regression coefficient, BMI = body mass index, CI = confidence interval, OR = odds ratio, TFQI = thyroid feedback quantile-based index, TSHI = TSH index, TT4RI = thyrotroph T4 resistance index.

**Table 6:** Separate age- and BMI-adjusted multivariable firth logistic regression models for hormone receptor status, HER2 status, and Ki-67 status.

Model	Thyroid index	*B*	Adjusted OR (95% CI)	*P* value
Panel A. HER2 status				
Model 1	TSHI	0.032	1.033 (0.457–2.334)	.939
Model 2	TT4RI	−0.004	0.996 (0.959–1.034)	.826
Model 3	TFQI	1.452	4.273 (0.575–31.768)	.156
Model 4	fT3/fT4 ratio	−1.489	0.226 (0.088–0.579)	**.002**
Panel B. Ki-67 status				
Model 1	TSHI	0.045	1.046 (0.557–1.966)	.888
Model 2	TT4RI	0.002	1.002 (0.973–1.032)	.896
Model 3	TFQI	−0.445	0.641 (0.135–3.039)	.575
Model 4	fT3/fT4 ratio	0.740	2.096 (1.219–3.603)	**.007**
Panel C. Estrogen receptor status				
Model 1	TSHI	0.248	1.281 (0.596–2.753)	.526
Model 2	TT4RI	0.013	1.014 (0.976–1.052)	.480
Model 3	TFQI	−0.044	0.957 (0.131–7.012)	.966
Model 4	fT3/fT4 ratio	−0.310	0.734 (0.416–1.293)	.284
Panel D. Progesterone receptor status				
Model 1	TSHI	−0.076	0.927 (0.470–1.826)	.826
Model 2	TT4RI	−0.003	0.997 (0.966–1.028)	.835
Model 3	TFQI	0.398	1.489 (0.272–8.142)	.646
Model 4	fT3/fT4 ratio	−0.201	0.818 (0.497–1.347)	.430

Bold values indicate statistically significant *P* values (*P* < .05).

Each adjusted estimate was obtained from a separate multivariable Firth penalized logistic regression model that included 1 thyroid index together with age and BMI. TSHI, TT4RI, TFQI, and the fT3/fT4 ratio were not entered simultaneously into the same model. Model 1 included TSHI; model 2, TT4RI; model 3, TFQI; and model 4, the fT3/fT4 ratio. Odds ratios represent a 1-unit increase in the respective thyroid index. No relevant multicollinearity was detected (maximum variance inflation factor = 1.11).

*B* = regression coefficient, BMI = body mass index, CI = confidence interval, OR = odds ratio, TFQI = thyroid feedback quantile-based index, TSHI = TSH index, TT4RI = thyrotroph T4 resistance index.

**Figure 2.**
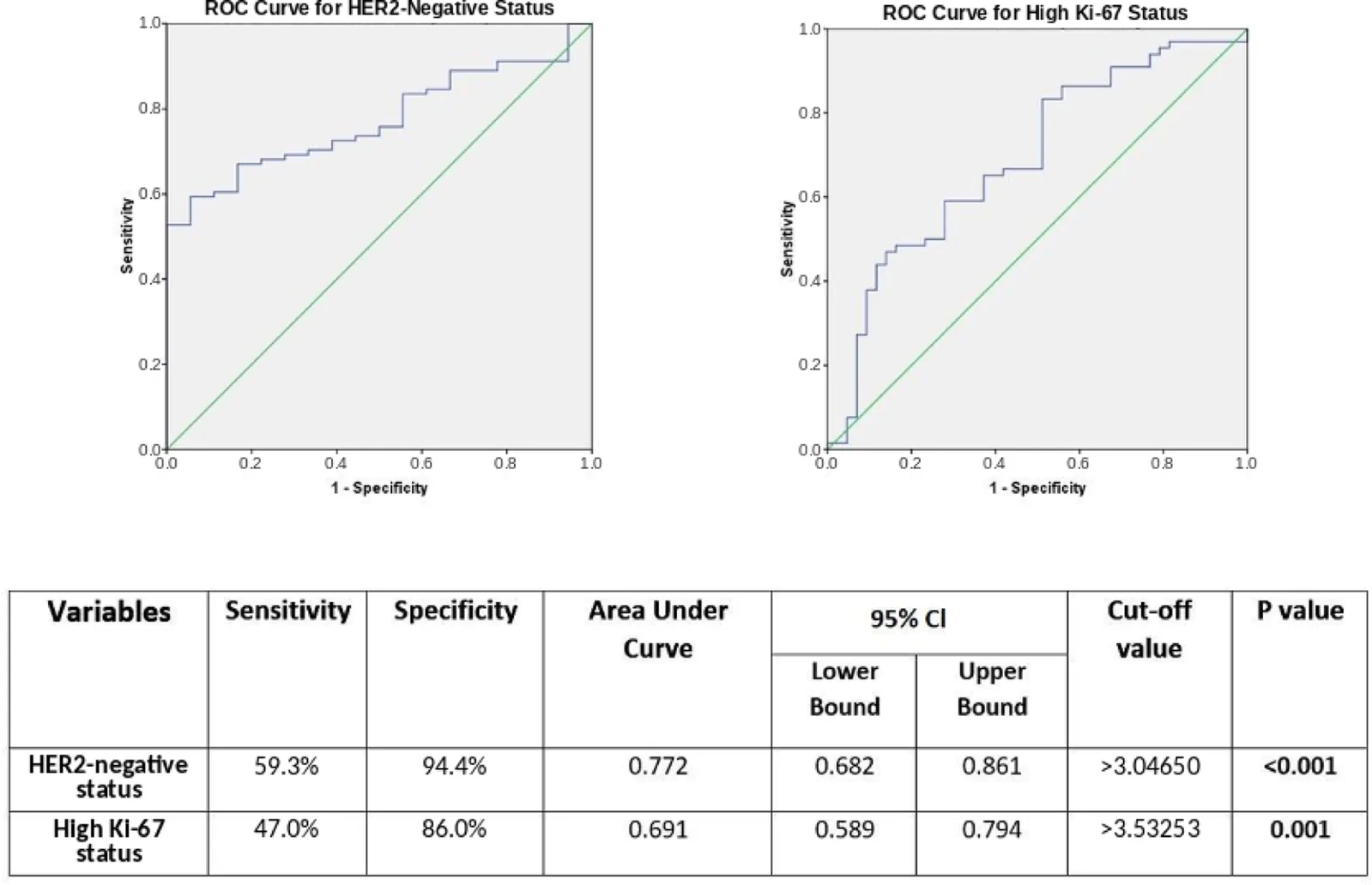
ROC-curve analysis of fT3/fT4 ratio for HER2 status and Ki-67 status. CI = confidence interval, fT3 = free triiodothyronine, fT4 = free thyroxine, ROC = receiver operating characteristic.

## 4. Discussion

Breast cancer has increasingly been recognized as a heterogeneous disease characterized by the interaction of histopathological, genetic, immunological, and molecular factors. The molecular classification described by Perou et al further highlighted this heterogeneity by identifying distinct subtypes of breast cancer with differences in prognosis and responses to treatment, particularly chemotherapy and hormone therapy. At present, the concept of breast cancer’s heterogeneity has expanded to include both intertumoral and intratumoral heterogeneity.^[15]^ Understanding the biological factors underlying intertumoral and intratumoral heterogeneity is important for explaining differences in tumor behavior and responses to treatment. In that context, we evaluated the associations of central thyroid hormone sensitivity indices and the fT3/fT4 ratio with surrogate clinicopathologic subtypes of breast cancer.

Although hormone levels are the most practical test used to show the activation of the endocrine gland responsible for a hormone, it is more useful to use certain indices, especially for the adrenal and thyroid glands, to determine the bioavailability of the hormone and negative or positive feedback. Whereas indices related to the adrenal system have long been used, thyroid system indices only entered the medical literature in the last 20 years. Moreover, although thyroid hormone sensitivity indices have increasingly been investigated in metabolic and oncological conditions, data regarding their relationships with surrogate clinicopathologic subtypes of breast cancer remain limited. To our knowledge, no previous study has specifically evaluated those indices in relation to the subtypes.

In our study, we first examined the association between the fT3/fT4 ratio and the surrogate clinicopathologic subtypes of breast cancer. Studies in the literature have examined the fT3/fT4 ratio and the coexistence of systemic diseases, and a rate increase has been found to be associated with metabolic diseases such as diabetes mellitus and obesity.^[16]^ However, the mechanisms of action remain unclear. In our results, the fT3/fT4 ratio differed across the surrogate clinicopathologic subtypes, with relatively higher values observed in the TNBC and luminal B-like/HER2-negative groups. To further explore the components underlying those subtype-based differences, we also evaluated the association of the fT3/fT4 ratio with markers used to define the surrogate clinicopathologic subtypes. Among the results, a higher fT3/fT4 ratio was associated with HER2-negative status and a high Ki-67 proliferation index, whereas no significant association was observed with ER or PR status. Those findings should be interpreted in the context of the prognostic relevance of the tumor characteristics. TNBC, for instance, is generally associated with an unfavorable prognosis compared with several other clinicopathologic subtypes. Furthermore, with the addition of a high Ki-67 proliferation index to triple-negative breast cancer, the coefficient of poor prognosis also increases.^[17,18]^ Several experimental studies have also shown thyroid hormone’s effect on the survival and proliferation of cancer cells. It was also found to have a similar effect to estradiol and the ability to suppress p53 and retinoblastoma proteins.^[19]^ However, while those results describe the relationship between thyroid hormone and breast cancer, the mechanisms underlying the observed associations with HER2 and Ki-67 remain incompletely understood.

An important finding of our study was that the associations of the fT3/fT4 ratio with HER2 status and the Ki-67 index persisted in separate Firth’s penalized logistic regression models adjusted for age and BMI. Each 1-unit increase in the fT3/fT4 ratio was associated with lower odds of HER2 positivity (adjusted OR = 0.226, 95% CI: 0.088–0.579; *P = *.002) and approximately twofold-higher odds of high Ki-67 (adjusted OR = 2.096, 95% CI: 1.219–3.603; *P = *.007). By contrast, the central thyroid hormone sensitivity indices did not show significant associations with those outcomes in the adjusted analyses. All in all, none of the mentioned findings establish causality or the underlying biological mechanism, and residual confounding cannot be excluded.

Albeit limited, studies investigating the relationship between thyroid hormone sensitivity indices and malignancy have largely focused on thyroid cancer. Following the introduction of TFQI by Laclaustra et al in 2019, those indices were initially investigated in the context of metabolic disorders and subsequently evaluated in oncological populations.^[8]^ In a study including 1998 patients with thyroid nodules, Sun et al reported that central thyroid hormone sensitivity indices, including TSHI, TT4RI, and TFQI, were positively associated with the risk of papillary thyroid carcinoma, whereas the fT3/fT4 ratio showed an inverse association and was also inversely correlated with TNM stage.^[20]^ In our cohort, however, we did not observe a significant association between central thyroid hormone sensitivity indices and the evaluated characteristics of breast cancer. Several factors may account for the difference. For one, our sample size may have limited our ability to detect relatively small effects of the central indices. For another, excluding patients with thyroid dysfunction or taking thyroid-related medication may have narrowed the distribution of TSH and consequently reduced the variability of TSH-based indices. Differences in target-organ biology should also be considered, because the direct trophic effects of TSH on thyrocytes are not applicable to breast tissue. Therefore, central thyroid axis indices may represent weaker markers in breast cancer than in thyroid malignancy.

Past research has suggested that the fT3/fT4 ratio may serve as an indirect proxy of the peripheral conversion of circulating T4 to T3, a process that involves the deiodinase enzymes D1, D2, and D3.^[21]^ Experimental studies have additionally shown alterations in deiodinase expression in tumor tissue and described the thyroid hormone-related activation of proliferative signaling pathways, including ERK1/2 and PI3K.^[22,23]^ However, because we did not measure deiodinase expression, local T3 availability, signaling pathway activity, and inflammatory mediators in our study, those mechanisms cannot be inferred from our data. A possible but untested hypothesis is that variation in peripheral thyroid hormone’s conversion may be associated with proliferative activity, which could provide context for the observed association between the fT3/fT4 ratio and Ki-67 status. Similarly, the lower fT3/fT4 ratio observed in HER2-positive patients might hypothetically reflect differences in tumor biology or systemic inflammatory responses. Those explanations remain speculative, however, and should be evaluated in studies incorporating direct tissue-based and molecular measurements.

In the models for ER and PR status, neither the fT3/fT4 ratio nor the central thyroid hormone sensitivity indices showed statistically significant associations. However, these findings do not establish the absence of an association, particularly given the limited sample size and precision of the estimates. Experimental studies have suggested that thyroid hormone may exert estrogen-like effects through ERK1/2-mediated activation of Erα, but our findings do not establish whether thyroid hormone sensitivity is associated with hormone receptor status.^[23]^ Furthermore, the associations observed with HER2 and Ki-67 should be interpreted as exploratory and hypothesis-generating because these analyses were not adjusted for multiple testing and may therefore be subject to false-positive findings. Confirmation in larger independent cohorts is needed.

Along with the fT3/fT4 ratio, we evaluated central thyroid hormone sensitivity indices in relation to the surrogate clinicopathologic subtypes of breast cancer. Past research has shown that the lower the TFQI value, the higher the sensitivity of the pituitary gland to thyroid hormone, and the higher the TT4RI and TSHI, the lower the central thyroid hormone sensitivity to thyroid hormone.^[7]^ In other words, the feedback mechanism has been interrupted. No statistically significant differences in the central thyroid hormone sensitivity indices were observed across the surrogate clinicopathologic subtypes. Studies have shown that reduced central sensitivity to thyroid hormone is associated with a lower risk of diabetes, as well as that increased central thyroid hormone sensitivity is directly proportional to obesity, renal failure, liver disease, and metabolic diseases.^[8]^ Although the mechanism has not been elucidated, all those relationships can be explained by the medial hormonal pathway network regulated by the central thyroid axis.^[24]^

Our findings have several limitations, primarily due to our study’s retrospective single-center design and relatively small sample size, which limited the scope of multivariable adjustment and subgroup analyses. Although we used Firth’s penalized logistic regression models and separate models adjusted for age and BMI, the small number of HER2-positive cases (n = 18) limited the precision of the estimates. Adjusting for age and BMI does not eliminate potential confounding by other clinical factors. Some potentially relevant variables, including menopausal status, complete tumor stage, and detailed medication use, were unavailable, and the ROC curve-derived cutoff values were not internally or externally validated. Therefore, causal inferences cannot be made, and our findings require confirmation in larger, preferably prospective cohorts. Despite those limitations, our findings provide a preliminary basis for further investigating the relationships between thyroid hormone sensitivity indices and surrogate clinicopathologic subtypes of breast cancer.

## 5. Conclusion

In our exploratory retrospective cohort study, the fT3/fT4 ratio differed across surrogate clinicopathologic subtypes of breast cancer and was associated with HER2 and Ki-67 status, whereas the central thyroid hormone sensitivity indices showed no significant differences. However, given the study’s single-center design, limited sample size, and potential residual confounding, those findings should be considered hypothesis-generating and require confirmation in larger prospective studies.

Supplementary Material, Supplemental Digital Content 1 is available for this article.

## Acknowledgments

We are grateful to all patients and doctors who contributed to this paper.

## Author contributions

**Data curation:** Ibrahim Burak Bahcecioglu.

**Formal analysis:** Sevket Baris Morkavuk.

**Methodology:** Safak Akin.

**Project administration:** Mehmet Ali Gulcelik.

**Supervision:** Nese Ersoz Gulcelik.

**Writing – review & editing:** Mehmet Mert Hidiroglu.

**Writing – original draft:** Sevket Baris Morkavuk.


