# Supplementary material for: Association between thyroid hormone sensitivity indices and surrogate clinicopathologic subtypes of breast cancer: A retrospective single-center study
Source: Medicine (Baltimore). 2026 Oct 30;105(44):e51340. doi: 10.1097/MD.0000000000051340 (PMC13727159; doi:10.1097/MD.0000000000051340)
Supplement: Supplementary file 1 [file medi-105-e51340-s001.docx]

**Supplementary Material** **1.** Conference abstract accepted for but subsequently withdrawn from the 18th St. Gallen International Breast Cancer Conference, 2023 (P028).
